# High-resolution respirometry in human endomyocardial biopsies shows reduced ventricular oxidative capacity related to heart failure

**DOI:** 10.1038/s12276-019-0214-6

**Published:** 2019-02-14

**Authors:** Daniel Scheiber, Tomas Jelenik, Elric Zweck, Patrick Horn, Heinz-Peter Schultheiss, Dirk Lassner, Udo Boeken, Diyar Saeed, Malte Kelm, Michael Roden, Ralf Westenfeld, Julia Szendroedi

**Affiliations:** 1 Division of Cardiology, Pulmonology and Vascular Medicine, Medical Faculty, Heinrich-Heine University, Düsseldorf, Germany; 2 Institute for Clinical Diabetology, German Diabetes Center, Leibniz Center for Diabetes Research, Heinrich Heine University, Düsseldorf, Germany; 3 German Center for Diabetes Research (DZD e.V.), München-Neuherberg, Partner Düsseldorf, Düsseldorf, Germany; 4 Institute for Cardiac Diagnostics and Therapy (IKDT), Berlin, Germany; 5 Clinic for Cardiovascular Surgery, Medical Faculty, Heinrich-Heine University, Düsseldorf, Germany; 6 Cardiovascular Research Institute Düsseldorf, Medical Faculty, Heinrich-Heine University, Düsseldorf, Germany; 7 Division of Endocrinology and Diabetology, Medical Faculty, Heinrich-Heine University, Düsseldorf, Germany

**Keywords:** Heart failure, Energy metabolism, Heart failure

## Abstract

The lifetime risk of developing heart failure is approximately 20%, and survival rates remain poor. Myocardial mitochondrial function has been suggested to play a pivotal role in heart failure pathophysiology. Human studies on ex vivo mitochondrial function have mostly been limited to atrial tissue obtained during open heart surgery and have provided contradictory results. This study aimed at measuring myocardial mitochondrial function in transcatheter ventricular endomyocardial biopsies and assessing the relationship between oxidative capacity and heart function. We enrolled 40 heart failure patients undergoing ventricular assist device surgery or heart transplantation (34 males, age 57 ± 11 years, body mass index 26.6 ± 4.8 kg/m^2^) and 29 heart transplant recipients of comparable age and body mass index with normal left ventricular function undergoing surveillance biopsies (23 males, 57 ± 12 years, body mass index 26.2 ± 4.1 kg/m^2^). High-resolution respirometry was established in the myocardium to measure oxidative capacity ex vivo. The mitochondrial oxidative capacity was 90% higher in ventricular compared to atrial tissues (*n* = 11, *p* < 0.01) of explanted hearts. Respiration rates were comparable in ventricular samples of heart failure patients obtained during open heart surgery by standard tissue preparation or ex vivo endomyocardial biopsy (*r* = 0.9988, *p* < 0.0001, *n* = 8), and the mitochondrial oxidative capacity in samples from these patients remained stable for 8 h when stored in either of two common preservation buffers. The oxidative capacity was 44% lower in heart failure than in transplant recipients (67 ± 3 vs. 97 ± 5 pmol/[s mg], *p* < 0.0001) and correlated positively with heart function (*r* = 0.49, *p* < 0.01). High-resolution respirometry of ventricular tissue is feasible in transcatheter biopsies, facilitating clinical studies on myocardial mitochondrial function in patients not undergoing heart surgery.

## Heart failure: Energy metabolism reflects cardiac function

The capacity of mitochondria in heart muscle cells to use oxygen to produce energy correlates with cardiac function. Julia Szendroedi at Heinrich-Heine University, Düsseldorf, Germany, and colleagues have established a technique to reliably evaluate mitochondrial energy metabolism in patients with or recovering from heart failure. They showed that the mitochondrial oxidative capacity of cells in the lower heart chambers (ventricles) was significantly higher than in the upper heart chambers (atria). Moreover, they found that mitochondrial oxidative capacity was reduced by 44% in heart muscle biopsies from patients with heart failure compared with biopsies from heart transplant recipients with normal ventricular function. Stimulating the respiration rate of mitochondria in ventricular heart cells could be a promising strategy for improving cardiac function.

## Introduction

Heart failure (HF) is a rising medical and societal burden^1^. Due to the aging of the population and improved survival from acute cardiac diseases, HF prevalence is increasing steadily, with a 20% lifetime risk for the development of HF^2^, which has a poor prognosis. The pathophysiology of HF is complex, but growing evidence from both preclinical and clinical studies points to impaired mitochondrial function in HF^3,4^. Thus, mitochondria appear to be a promising therapeutic target to improve cardiac function^5^.

Current in vivo noninvasive measurement of heart energy metabolism relies on the relative concentrations of phosphorous metabolites and is restricted to selected clinical centers^6–8^. Polarographic measurement of ex vivo mitochondrial oxygen consumption in the presence of specific substrates has been widely accepted as the gold-standard method for analyzing mitochondrial oxidative capacity and efficiency in human tissue samples, i.e., skeletal muscle and the liver^9–13^. Thus far, assessment of ex vivo cardiac mitochondrial respiration has been limited to samples acquired during open heart surgery, which precludes follow-up measures during clinical trials and restricts patient groups. Of note, the mitochondrial oxidative capacity of left ventricular (LV) tissue obtained during open heart surgery was decreased by 40% in terminal HF patients compared to patients with an LV ejection fraction >45%^14^.

Human transcatheter endomyocardial biopsies (EMBs) of the interventricular septum (IVS) are routinely applied for the management of cardiovascular diseases according to the latest scientific statement of the American Heart Association, the American College of Cardiology, and the European Society of Cardiology^15^. However, EMB has not yet been employed to assess mitochondrial function.

A number of studies analyzed atrial tissue in patients undergoing valve replacement or coronary artery bypass surgery^16,17^, referred to as the “control group”, while analyses of ventricle tissue were mostly applied to patients with end-stage HF following heart transplantation or left ventricular assist device (LVAD) surgery^14,18^. Meanwhile, accumulated evidence suggests that the atrial mitochondrial oxidative capacity does not reflect that of ventricular tissue; thus, comparisons of different patient groups must address corresponding heart areas^17,18^. Moreover, the use of mitochondrial preservation medium^19^ or standard tissue perfusion solution^17^, as well as the storage time until mitochondrial respiration analysis, might also affect respiration rates. Taken together, standardized procedures applicable to clinical trials in larger cohorts are warranted.

To this end, we analyzed mitochondrial respiration in right atrial, LV, and IVS tissue specimens from heart explants. We then established high-resolution respirometry (HRR) in human transcatheter EMB of the IVS. We tested for the impact of the time lag between tissue acquisition and the start of the experiment and the effect of different preservation media on respiration measurements. Finally, we tested the clinical applicability in a cohort of heart transplant recipients with normal LV function and in patients with terminal HF and related mitochondrial respiration rates to heart function.

## Materials and methods

### Study protocol

This registered clinical trial protocol was approved by the ethics board of the university hospital of the Heinrich-Heine University Düsseldorf (Study Number: 5263R; ClinicalTrial.gov registration number NCT03386864). All procedures conformed to the World Medical Association Declaration of Helsinki. Before inclusion, all participants gave written informed consent.

### Study participants and tissue acquisition

**Fig. 1:**
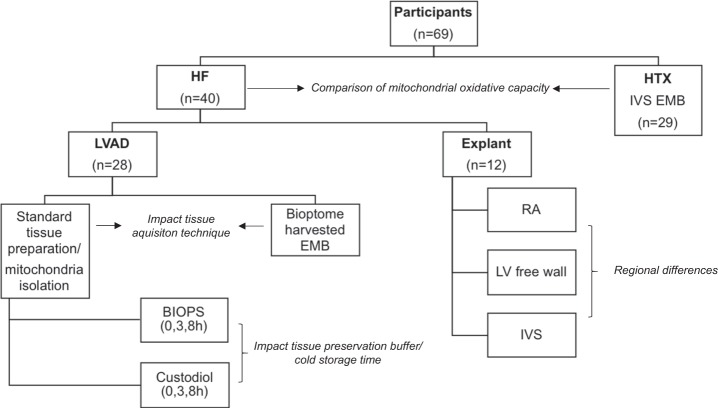
Patient characteristics. The mitochondrial oxidative capacities in tissue specimens of HF patients and heart transplant recipients was compared. To assess the impact of the tissue acquisition technique on mitochondrial respiration, standard prepared tissues and ex vivo bioptome-harvested endomyocardial biopsies were compared to tissue specimens obtained during LVAD surgery. The impact of the tissue preservation buffer and cold storage time on mitochondrial respiration was analyzed in standard prepared tissues and isolated mitochondria from tissue specimens obtained during LVAD surgery. Heart explant tissue specimens were obtained from the RA, LV free wall, and IVS to analyze regional differences in mitochondrial respiration. HF patients suffering from terminal heart failure, HTX heart transplant recipients, IVS interventricular septum, LV left ventricle, LVAD left ventricular assist device, RA right atrium

Myocardial tissue specimens were obtained from a total of 69 individuals (Fig. 1). In the HF group (HF, *n* = 40), tissue specimens were obtained surgically during LVAD implantation (*n* = 28) or heart explantation (*n* = 12). In stable heart transplant recipients with a normal LV function (HTX, *n* = 29), tissue specimens were obtained during routine surveillance EMB (Fig. 1).

#### LVAD surgery

During LVAD surgery, the part of the cardiac apex that corresponds to the LV was excised (Supplementary Figure 1a), separated into two pieces, and immediately transferred to ice-cold preservation medium (BIOPS; containing [in mM] 2.77 CaK_2_EGTA, 7.23 K_2_EGTA, 20 imidazole, 20 taurine, 50 4-morpholine-ethanesulfonic acid, 0.5 dithiothreitol, 6.56 MgCl_2_·6H_2_O, 5.77 Na_2_ATP, and 15 disodium phosphocreatine) or ice-cold CUSTODIOL^®^ perfusion solution containing [in mM] 15 sodium chloride, 9.0 potassium chloride, 1 potassium hydrogen 2-ketoglutarate, 4.0 magnesium chloride, 18.0 histidine HCl, 180.0 histidine, 2.0 tryptophan, 30.0 mannitol, and 0.015 calcium chloride in sterile water. Samples were transferred to the laboratory in ice-cold buffer within a few minutes. Part of the ventricular tissue was directly prepared for HRR. The remaining tissue was kept on ice in BIOPS or CUSTODIOL^®^ for 3 or 8 h to assess the impact of storage buffer and time on respirometry. To assess the impact of sample size and tissue acquisition technique on mitochondrial respiration, we harvested EMBs ex vivo using an EMB bioptome from tissue samples of eight patients obtained during LVAD surgery and compared the mitochondrial respiration in these EMBs to that in standard preparations of tissue specimens of the same patients using scissors and forceps.

#### Heart transplantation

Specimens of explant hearts were harvested from the right atrium, the LV free wall, and the IVS immediately after explantation and transferred to ice-cold BIOPS preservation solution (Supplementary Figure 1b).

#### Endomyocardial biopsy

Heart transplant recipients underwent surveillance EMB as a part of the routine allograft rejection monitoring. All EMB procedures were performed via right femoral vein access^20^. After placing a sheath across the tricuspid valve into the right ventricle pointing towards the right IVS (Supplementary Figure 1c), four to five biopsy samples were taken and further processed according to clinical routine, while two additional samples were immediately transferred to ice-cold BIOPS. The mitochondrial respiration in IVS EMBs of heart transplant recipients was compared to the mitochondrial respiration in LV tissue specimens of HF patients undergoing LVAD surgery or heart transplantation. Only EMBs of patients without histological signs of acute cellular or humoral transplant rejection were included in the study.

### Oxygraph measurements

#### Mitochondrial respiration

Myocardial mitochondrial respiration in permeabilized myocardial muscle fibers and isolated mitochondria was analyzed ex vivo using HRR following procedures previously established and/or developed for skeletal muscle and liver tissue (OROBOROS Oxygraph-2k, Austria)^9–11^. Citrate cycle-derived substrates and the medium-chain fatty acid octanoyl-carnitine were applied^9,21^. Samples obtained during LVAD surgery or heart transplantation were prepared from intact and scar-free areas of the myocardium. Myocardial fibers were carefully dissected, permeabilized in BIOPS supplemented with 50 µg/ml saponin (Sigma-Aldrich Chemie GmbH, Germany) for 30 min, and then washed in respiration medium (MiRO5; containing [in mM] 0.5 EGTA, 3 MgCl_2_·6H_2_O, 60 K-lactobionate, 20 taurine, 10 KH_2_PO_4_, 20 HEPES, 110 sucrose, and 1 g/l bovine serum albumin, essentially fatty acid free in sterile water)^22^. Thereafter, tissue specimens were blotted on filter paper, weighed, and transferred to oxygraph chambers containing 2.3 ml MiRO5. Respiration was measured at 37 °C at oxygen concentrations between 120 and 450 µM in duplicate and analyzed using Datlab software (OROBOROS Instruments Corp., Innsbruck, Austria). Oxygen fluxes represent basal or substrate-saturated respiration before adding ADP, ADP-stimulated coupled respiration with octanoyl-carnitine, glutamate, and succinate (state III), and maximal uncoupled respiration after adding the uncoupling factor fccp (state u)^23^. The oxidative capacity of the mitochondrial oxidative phosphorylation system (OXPHOS capacity) was derived from state III respiration after addition of octanoyl-carnitine, glutamate, and succinate. Mitochondrial leak respiration (state 4o) was induced through inhibition of the enzyme ATP synthase by adding oligomycin^12^. The respiratory control ratio (state III/state 4o) and the leak control ratio (state 4o/state u) were calculated as surrogate markers of mitochondrial coupling efficiency^9,12^. The integrity of the outer mitochondrial membrane was analyzed via exogenous stimulation of state III respiration by cytochrome *c*^24^. A stimulation of oxygen flux less than 10% after cytochrome *c* addition is considered to indicate a preserved outer mitochondrial membrane^24^.

#### Citrate synthase activity

The mitochondrial content was assessed from the citrate synthase activity (CSA) as described previously^25,26^. In short, CSA was measured spectrophotometrically in lysed cells at a 412 nm wavelength after addition of 10 mM dithionitrobenzoic acid, 30 mM acetyl-CoA, and 10 mM oxaloacetic acid. The results have been normalized to the protein content measured colorimetrically via bicinchoninic acid assay.

#### Fluoroscopic analysis of hydrogen peroxide emission

Fluoroscopic measurements of mitochondrial hydrogen peroxide release were performed using an Oxygraph-2k combined with the Fluorescence-Sensor Green of the O2k-Fluo LED2-Module for hydrogen peroxide (OROBOROS Instruments, Austria)^10,26^. An aliquot of tissue sample prepared for the HRR measurements was transferred to oxygraph chambers containing 2.3 ml MiR5 with 10 mM of the extrinsic fluorophore Amplex red (Thermo Fisher Scientific, USA) and horseradish peroxidase (1 U/ml; Sigma-Aldrich Chemie GmbH, Germany)^27^. Catalyzed by horseradish peroxidase, Amplex red, and hydrogen peroxide produce the red fluorescent compound resorufin (excitation wavelength 563 nm and emission 587 nm). The change of the emitted fluorescence intensity during stepwise addition of the mitochondrial substrates malate and succinate is directly proportional to the production of hydrogen peroxide^28^. Calibrations were performed with a standard hydrogen peroxide addition.

#### Isolated mitochondria

Mitochondria were isolated by differential centrifugation as described previously^9^. Oxygraph measurements of isolated mitochondria were performed following the same protocol as for permeabilized fibers, but in a different respiration buffer (MiRO1; containing [in mM] 100 sucrose, 100 KCl, 20 TES, 4 KH_2_PO_4_, 1 EDTA, 2 MgCl_2_·6 H_2_O, and 1 g/l bovine serum albumin, essentially fatty acid free in sterile water) at saturating oxygen levels.

### LV function

The LV ejection fraction was analyzed using the modified biplane Simpson’s rule according to current European Society of Cardiology HF guidelines with a Philips HD15 ultrasound system (Philips Healthcare, The Netherlands)^29^. The cardiac index was calculated using a modified Fick method during right heart catheterization at the time of EMB or at the time of evaluation for heart transplantation and/or LVAD implantation^30^.

### Gene expression analyses

Relative quantification of myocardial messenger ribonucleic acid (mRNA) expression of genes related to mitochondrial respiratory chain complex I (NDUFS1) and an important mitochondrial transport enzyme, carnitine palmitoyltransferase 1B (CPT1B), was assessed with real-time PCR (QuantiTect Reverse Transcription Kit; Qiagen) and the comparative threshold cycle method (DDCt) with 18S ribosomal RNA as the reference gene, as described previously, in tissue specimens of a small subset of HF patients undergoing LVAD surgery and heart transplant recipients undergoing EMB^9^.

### Statistical analyses

Statistical analyses were performed using Prism 7 (Version 7.0a; GraphPad Software, Inc., USA) and SPSS (Version 24.0.0.2; IBM SPSS Statistics, USA). Data are expressed as the means ± standard deviation or means ± standard error of the mean. *P*-values ≤0.05 were considered significant. Data were analyzed using the paired or unpaired Student's *t*-test, two-way analysis of variance (ANOVA), and the Sidak post hoc test. Datasets with no Gaussian distribution were analyzed using the Mann–Whitney *U* test. To test for a linear association between two variables, we used Pearson or Spearman correlation models and partial correlation, depending on whether data were normally distributed.

## Results

### Patients' characteristics

The distribution of age, sex, and body mass index was comparable in HF patients and heart transplant (HTX) recipients (Table 1). Table 1 provides an overview of the comorbidities, cardiac status, and laboratory data of patients in both groups. All HTX patients received an immunosuppressive combination treatment consisting of prednisolone, tacrolimus, mycophenolate mofetil, and/or everolimus (Supplementary Table 1). Cardiac function in HF patients was significantly impaired compared to HTX patients, with an approximately 60% lower LV ejection fraction and an approximately 40% lower cardiac index. HF was associated with increased CRP, while HTX patients had elevated HbA1c serum levels.

**Table 1:** Patient characteristics

	HF (LVAD/explant) (*n* = 40)	HTX (*n* = 29)	*p* Value
**Anthropometry**			
Sex [% male]	85	79	0.75
Age [years]	57 ± 11	57 ± 12	0.93
BMI [kg/m^2^]	26.6 ± 4.8	26.2 ± 4.1	0.69
**Comorbidities**			
T2DM [%]	28	38	0.44
Terminal CKD [%]	13	10	>0.99
Arterial hypertension [%]	45	38	0.63
COPD [%]	20	10	0.34
**Cardiac characteristics**			
EF [%]	26.1 ± 16.6	66.3 ± 6.9	<0.0001
Cardiac index [l/min/m²]	1.7 ± 0.3	2.9 ± 0.6	<0.0001
Time post-HTX [months]	–	15.8 ± 7.1	–
ICM [%]	55	38	0.22
DCM [%]	45	66	0.14
**Lab values**			
Creatinine [mg/dl]	1.5 ± 0.7	1.3 ± 0.5	0.37
CRP [mg/dl]	4.4 ± 6.0	1.3 ± 3.6	0.0006
LDL [mg/dl]	90 ± 38	106 ± 28	0.10
HbA1c [%]	5.7 ± 0.6	6.3 ± 1.0	0.02
HbA1c [mmol/mol]	38.3 ± 7.0	45.1 ± 11.2	0.02

*BMI* body mass index, *CKD* chronic kidney disease, *COPD* chronic obstructive pulmonary disease, *CRP* *C-reactive protein*, *DCM* dilated cardiomyopathy, *EF* left ventricular ejection fraction, *HF* heart failure, *HTX* heart transplantation, *ICM* ischemic cardiomyopathy, *LDL* low-density lipoprotein, *LVAD* left ventricular assist device, *T2DM* diabetes mellitus type 2.

Means ± SD; *p*-values calculated with the unpaired *t*-test/Mann–Whitney test

### Regional differences in myocardial mitochondrial respiration

**Fig. 2:**
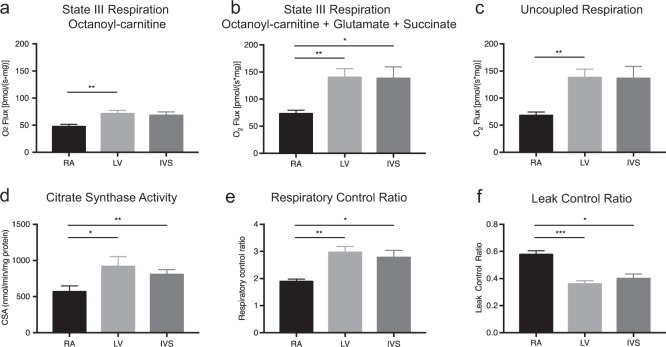
Comparison of myocardial right atrial, left ventricular, and IVS tissue specimens obtained from heart explants. **a** State III mitochondrial respiration on the medium-chain fatty acid octanoyl-carnitine. **b** State III mitochondrial respiration on octanoyl-carnitine and the citrate cycle-derived substrates glutamate and succinate. **c** Uncoupled mitochondrial respiration after addition of the uncoupling factor fccp (carbonyl cyanide-*4*-(trifluoromethoxy)phenylhydrazone). **d** Citrate synthase activity per milligram protein as a marker of mitochondrial content. **e** Respiratory control ratio as a marker of increased mitochondrial coupling efficiency. **f** Leak control ratio as a marker of impaired mitochondrial coupling efficiency. A atrium, CSA citrate synthase activity, IVS interventricular septum, L left, R right, V ventricle. Data are the means ± SEM (*n* = 9–11/group). Multiple paired *t*-test with Bonferroni correction. *p < 0.05; **p < 0.01

Tissue specimens of explant hearts were harvested from the right atrium, the LV free wall, and the IVS of HF patients. Compared to right atrial tissue, specimens from the LV free wall and the IVS revealed 50% increased state III myocardial respiration on octanoyl-carnitine (RA: 47.7 ± 4 vs. LV: 71.3 ± 6.2 vs. IVS: 68.3 ± 6.5 pmol/[s mg]; *p* < 0.01). After addition of the citrate cycle-derived substrates glutamate and succinate, mitochondrial respiration in ventricular samples was approximately 90% higher (RA: 73.2 ± 6.4 vs. LV: 140.2 ± 15.8 vs. IVS: 138.4 ± 21 pmol/[s mg]; *p* < 0.01) (Fig. 2a, b). The uncoupled oxygen flux doubled in LV compared to atrial tissue specimens (Fig. 2c). CSA was approximately 60% higher in LV tissue specimens compared to atrial samples (RA: 570 ± 79 vs. LV: 917 ± 138 vs. IVS: 806 ± 68 nmol/min/mg protein; *p* < 0.01) (Fig. 2d). The respiratory control ratio was increased by 52% and the leak control ratio was decreased by 34% in ventricular tissue compared to atrial tissue, indicating higher coupling efficiency in ventricular tissue (Fig. 2e, f).

### Impact of the sample size and tissue acquisition technique on mitochondrial respiration

**Fig. 3:**
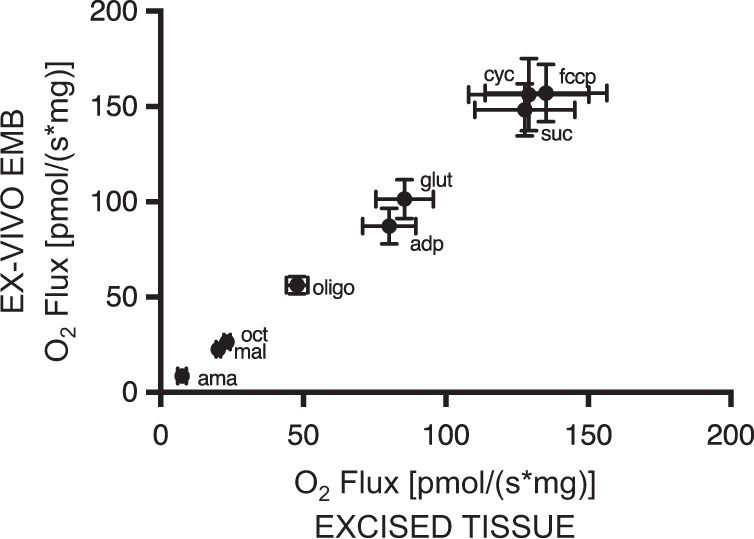
Mitochondrial state III respiration of biopsies endoscopically harvested using a bioptome revealed the same results compared to standard excised myocardial tissue specimens from left ventricular apex tissue samples obtained during left ventricular assist device surgery. Mitochondrial respiration was measured after addition of octanoyl-carnitine and the citrate cycle-derived substrates glutamate and succinate in an oxygraph. ama antimycin A, mal malate, oct octanoyl-carnitine, oligo oligomycin, adp adenosine diphosphate, glu glutamate, suc succinate, cyc cytochrome *c,* fccp carbonyl cyanide-*4*-(trifluoromethoxy)phenylhydrazone. Pearson correlation; data are the means ± SEM (*n* = 8/group)

There was a strong correlation between oxygen fluxes measured in EMBs harvested using a bioptome from ventricular apex tissue samples obtained during LVAD surgery of HF patients and fluxes measured after standard tissue preparation for the same tissues (*r* = 0.9988, *p* < 0.0001) (Fig. 3). In tissues obtained using both acquisition techniques, oxygen consumption rates with both octanoyl-carnitine and the citrate cycle-derived substrates glutamate and succinate were increased by less than 10% after addition of cytochrome *c*, indicating stable conditions with an intact outer mitochondrial membrane (standard preparation: 135.2 ± 21.4 [+5.5%] vs. EMB: 157.1 ± 15 pmol/[s mg] [+6.1%]).

### Impact of the cold storage time on mitochondrial respiration

In nine ventricular apex tissue specimens of LVAD patients from the HF group (Table 1), HRR was performed repetitively after 0, 3, and 8 h of cold storage time in BIOPS or CUSTODIOL^®^ preservation buffer. These measurements displayed stable oxygen flux rates for each timepoint and buffer in mitochondrial state III respiration on solely octanoyl-carnitine and the citrate cycle-derived substrates glutamate and succinate plus octanoyl-carnitine, with no effect on uncoupled respiration or the respiratory control ratio (Supplementary Figure 2). Oxygen flux was also measured in mitochondria isolated from the ventricular apex of the same patients, representing the intrinsic mitochondrial function detached from the surrounding tissues. The measurements showed that the use of different preservation media did not affect the oxygen flux rates in isolated mitochondria (Supplementary Figure 3). Assessment of hydrogen peroxide emission on the respiratory chain substrates malate and succinate revealed no differences in mitochondrial peroxide production after 0, 3, and 8 h of cold storage in BIOPS tissue preservation media (Supplementary Figure 4a). In tissue specimens stored for 8 h in ice-cold CUSTODIOL^®^ tissue preservation media, mitochondrial peroxide emission was significantly increased compared to 0 and 3 h of cold storage in CUSTODIOL^®^ and compared to all time points of BIOPS storage (*p* < 0.01) (Supplementary Figure 4a, b).

### Mitochondrial oxidative capacity in HF patients and HTX patients with a normal LV function

**Fig. 4:**
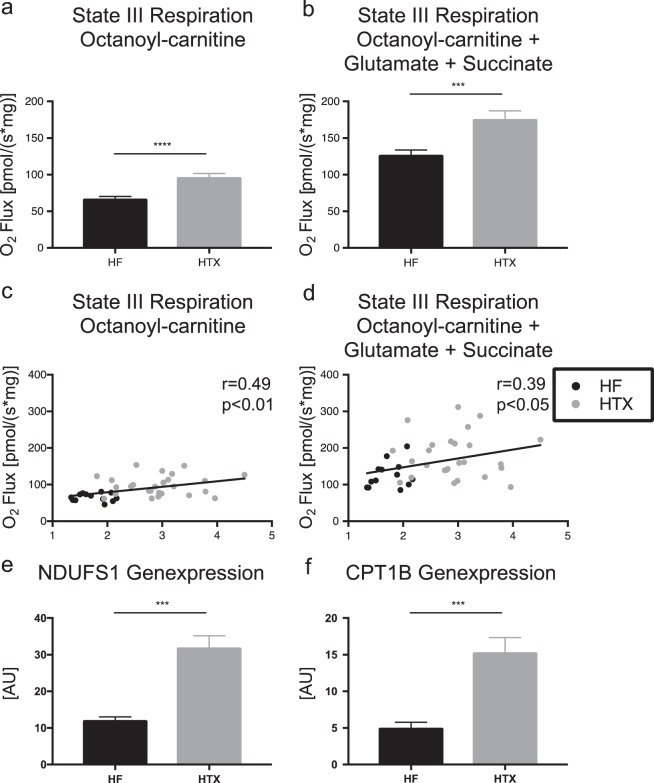
State III myocardial respiration on octanoyl-carnitine (**a**, **c**) and on the citrate cycle-derived substrates glutamate and succinate (**b**, **d**) and mRNA expression of NDUFS1 and CPT1B in myocardial tissue specimens from patients suffering from terminal heart failure and heart transplant recipients. **a**, **b** Comparison of maximal myocardial respiration. Student's *t*-test. **c**, **d** Correlation of maximal myocardial respiration and cardiac index measurements. Spearman correlation. **e**, **f** Expression of NDUFS1 and CPT1B mRNA. Student's *t*-test. *n* = 40 (HF, patients suffering from terminal heart failure; *n* = 4 in mRNA measurements), *n* = 29 (HTX, heart transplant recipients; *n* = 21 in mRNA measurements). AU arbitrary unit. ***p < 0.001, ****p < 0.0001

Based on the above-mentioned experiments, the mitochondrial respiration in LV and IVS tissue specimens is comparable. In EMBs endoscopically harvested from the IVS of heart transplant recipients with a normal LV function, state III mitochondrial respiration on the medium-chain fatty acid octanoyl-carnitine was 44% (67.4 ± 3.0 vs. 96.9 ± 4.80 pmol/[s mg], *p* < 0.0001) higher and, after addition of the citrate cycle-derived substrates glutamate and succinate, 38% (127.4 ± 6.4 vs. 176.2 ± 10.8 pmol/[s mg], *p* < 0.0001) higher compared to LV tissue specimens of HF patients obtained during open heart surgery (Fig. 4a, b). Oxygen flux measurements on octanoyl-carnitine and after addition of the citrate cycle-derived substrates glutamate and succinate correlated positively with cardiac index measurements in terminal HF patients and heart transplant recipients (octanoyl-carnitine: *r* = 0.49, *p* < 0.001; octanoyl-carnitine + glutamate + succinate: *r* = 0.39, *p* < 0.05) (Fig. 4c, d), even after correction for HbA1c serum concentrations (octanoyl-carnitine: *r* = 0.46, *p* = 0.006; octanoyl-carnitine + glutamate + succinate: *r* = 0.39, *p* = 0.024; data not shown).

### Gene expression in HF patients and HTX patients with a normal LV function

Expression of NDUFS1 (12.18 ± 0.83 vs. 32.01 ± 3.13[AU], *p* < 0.0001) and CPT1B (5.04 ± 0.74 vs. 15.36 ± 1.98[AU], *p* = 0.0006) mRNA was significantly reduced in LV tissue specimens of HF patients undergoing LVAD surgery compared to IVS EMBs of HTX patients with a normal LV function (Fig. 4e, f).

## Discussion

In this study, we established HRR in human EMB of the IVS and tested its clinical applicability. Ventricular tissue specimens had markedly increased oxidative capacity, coupling efficiency, and mitochondrial content compared to atrial tissue samples. Cold storage for up to 8 h in the most commonly used preservation media did not affect oxygen flux measurements in human IVS EMBs. The mitochondrial oxidative capacity of LV tissue was reduced by 44% in HF compared to HTX patients and correlated positively with the cardiac index. Thus, HRR is feasible and robust in human EMB of the IVS, facilitating clinical studies addressing heart mitochondrial function in ventricular tissue of HF patients without restriction to open heart surgery.

### Increased oxidative capacity in ventricular compared to atrial tissue specimens

Analysis of state III myocardial respiration in LV, IVS, and right atrial tissue specimens obtained from explant hearts of HF patients revealed increased mitochondrial oxygen flux in ventricular compared to atrial tissue specimens. The ventricular OXPHOS capacity in our study was two-fold higher compared to atrial tissues, in line with previous results showing a factor of 2.3 between atrial and ventricular tissues in explant hearts of patients suffering from ischemic or dilated cardiomyopathy^17,18^. We compared mitochondrial respiration in tissue samples taken from the right atrium, LV free wall, and IVS. Ventricular tissue specimens harvested from the LV free wall or IVS showed comparable mitochondrial respiration. This is an important finding, as EMBs are mostly harvested from the IVS via jugular or femoral vein access and not from the LV free wall in clinical routine^31^. Our results highlight the importance of tissue acquisition for evaluation of myocardial energy metabolism since ventricular tissue reflects cardiac function rather than atrial tissue^16,32^. Previous studies analyzed regional differences in mitochondrial respiration in heart explants for only a small number of cases (*n* = 3)^17^ or did not focus on differences in mitochondrial respiration between IVS and LV tissue specimens.^18^ To the best of our knowledge, this is the first study analyzing regional differences in mitochondrial respiration for a significant number of cases (*n* = 9–11). Increased ventricular OXPHOS capacity compared to atrial tissues might relate to higher mitochondrial content, as suggested by CSA measurements, but calculation of the respiratory control ratio and leak control ratio as surrogate markers of mitochondrial coupling efficiency^9,12^ suggests qualitative differences in mitochondrial respiration between ventricular and atrial tissues.

### Bioptome-harvested EMBs show equivalent results to samples obtained with the surgical tissue acquisition technique

We analyzed mitochondrial respiration in myocardial samples excised by a scalpel compared to biopsies harvested from the same sample using an endomyocardial bioptome. Both tissue acquisition techniques revealed equal oxygen flux rates. Cytochrome *c* addition resulted in no relevant increase in O_2_ flux in all measurements, indicating outer mitochondrial membrane integrity^33^. In contrast to earlier studies, where fiber bundles from 4–6 mg^22^ to 10–40 mg^17^ were obtained during heart transplantation^18^, LVAD, or heart valve surgery^14,16,34^, EMBs taken by a bioptome in our study had a tissue weight of at most 0.5–2 mg. These results suggest that the mechanical stress exerted by a bioptome and differences in tissue weight do not impact oxygen flux measurements compared to standard tissue preparation. To the best of our knowledge, this is the first study analyzing gold-standard myocardial mitochondrial oxidative phosphorylation in bioptome-harvested ventricular EMBs from humans. This facilitates the possibility of including samples from patients in mitochondrial respiration analysis that would not be accessible by a surgical approach. These patients comprise all patients with a clinical indication for EMB. According to the latest scientific statements of the American Heart Association, the American College of Cardiology, the European Society of Cardiology and the International Society for Heart and Lung Transplantation, these include especially patients with new onset of HF with hemodynamic compromise, HF patients with an unexplained restrictive cardiomyopathy, and heart transplant recipients undergoing routine surveillance EMB to test for heart transplant rejection^15,35^. Especially in heart transplant recipients, scheduled follow-up EMB facilitates the possibility of longitudinal analysis of myocardial mitochondrial respiration under various clinical influences. Consequently, a great number of patients with various pathologies are becoming accessible for HRR analysis, providing the opportunity for a deeper understanding of myocardial mitochondrial energy metabolism.

### Stable mitochondrial respiration independent of preservation buffer and storage time

To analyze the impact of the cold storage time and preservation buffer on heart energy metabolism, we assessed respiration and ROS production in tissue specimens obtained during LVAD surgery. The mitochondrial state III respiration on fatty acids and citrate cycle-derived substrates, maximal uncoupled respiration, and coupling efficiency were comparable for the two most widely used preservation media, BIOPS and CUSTODIOL^®^, independent of cold storage time up to 8 h. This corresponds to findings in surgically assessed human heart samples showing no changes in respiratory parameters during the first 9 h of tissue preservation before membrane defects occurred^17^. Cardioplegic solutions and BIOPS have previously been used as a storage buffer in studies analyzing myocardial mitochondrial function^18,34,36^, but to the best of our knowledge, there are no studies comparing mitochondrial respiration after storage in different solutions. We compared the mitochondrial preservation abilities of BIOPS with CUSTODIOL^®^, an organ preservation solution frequently used for heart transplantation in Europe^37,38^. While respiratory parameters were similar, the mitochondrial peroxide release increased after 8 h of CUSTODIOL^®^ storage compared to BIOPS at all time points. This comparison demonstrates the comparability of different study protocols and buffers used in previous studies and suggests the preferential usage of BIOPS for tissue preservation, especially if a delay in measurement initiation is expected and ROS formation is analyzed.

### Decreased myocardial OXPHOS capacity in terminal HF patients

The mitochondrial OXPHOS capacity in terminal HF patients was reduced 44% compared to HTX recipients with a normal LV function. In contrast to previous studies comparing mitochondrial respiration in surgically obtained tissue specimens of patients with coronary vs. valvular heart disease^16^, LVAD vs. valvular surgery^14,34^, and failing explant vs. donor hearts^17,18^, we not only detected differences in mitochondrial energy metabolism between HF and control patients but also, for the first time, were able to show a significant correlation of mitochondrial respiration and CI measurement, highlighting a direct and close link of energy metabolism to cardiac function. This correlation over a large number of patients remained significant, even after correction for HbA1c serum concentrations, underlining the profound connection of mitochondrial respiration and CI, considering that there is some evidence for impaired mitochondrial respiration in myocardial tissue specimens of patients suffering from type 2 diabetes mellitus^39^.

Our results support previous data showing 40% diminished OXPHOS capacity in LV tissue specimens of patients undergoing LVAD surgery compared to LV tissue specimens of control individuals with good LV function undergoing cardiac valve surgery^14^. In contrast, analysis of myocardial mitochondrial respiration in isolated mitochondria from LV tissue specimens revealed no differences in OXPHOS capacity between terminal HF and control patients^19,36^, possibly indicating that the intrinsic mitochondrial capacity is not affected.

A major challenge of studies analyzing human myocardial mitochondrial respiration in terminal HF is the comparison of pathologic findings with an appropriate control group. It is well known that pressure and volume overload impact myocardial oxygen consumption and energy metabolism; thus, patients with valvular heart surgery might not serve as an adequate control group for patients suffering from HF^40^. Additionally, nonfailing explant hearts from patients undergoing combined heart-lung transplantation following severe pulmonary hypertension experience pressure overload and thus do not correspond to healthy hearts. Donor hearts that were not eligible for transplantation or could not be allocated also have limitations for use as controls, as these organs have a long cold ischemia time that affects transplant integrity^41^. Therefore, defining an adequate control group is one of the main limitations in analyzing human heart tissue. Here, we established HRR for the analysis of mitochondrial respiration in endoscopically harvested EMBs. Analyzing EMBs enables us to obtain tissue specimens independent of cardiac surgery and increase case numbers for a better understanding of mitochondrial respiration in HF pathophysiology.

### Decreased myocardial mitochondrial biogenesis mRNA expression in HF patients

In a murine model of induced HF, decreased expression of genes related to mitochondrial biogenesis, e.g., PGC-1α, has been shown^42^. Information on mitochondrial biogenesis in human HF conflicts with the murine results. The amount of mRNA for subunits of the mitochondrial electron transfer chain is normal in explanted failing hearts compared with donor hearts^43^. Our results support an HF-associated decline in the expression of genes associated with mitochondrial respiration. Further studies focusing on gene expression are needed to address this issue.

### Limitations

According to previous studies in the field, defining a control group is challenging. We compared energy metabolism in terminal HF patients with HTX recipients. These patients were characterized by a normal cardiac index and no signs of allograft rejection but received an immunosuppressive therapy whose impact on mitochondrial OXPHOS capacity is unknown. However, our correlation of cardiac index measurements with myocardial OXPHOS capacity showed robust results over all patients, as well as within the HF or post-HTX group.

## Conclusion

In this study, we established HRR as a gold-standard method for the analysis of mitochondrial respiration in human EMB. We detected local differences in mitochondrial respiration, with markedly increased oxidative capacity in ventricular compared to atrial tissue specimens. HRR measurements generated robust results even after prolonged cold storage. Application of this technique in a clinical setting showed a 44% reduced myocardial OXPHOS capacity in HF compared to HTX patients, with a positive correlation of mitochondrial oxygen respiration to cardiac function. This technique will facilitate evaluation of mitochondrial energy metabolism in various patient cohorts, helping to decipher the pathophysiological impact of a disturbed myocardial energy metabolism on HF.

## Supplementary information

Supplementary Table 1

Supplementary Figure 1

Supplementary Figure 2

Supplementary Figure 3

Supplementary Figure 4
