# Supplementary material for: High-resolution respirometry in human endomyocardial biopsies shows reduced ventricular oxidative capacity related to heart failure
Source: Exp Mol Med. 2019 Feb 14;51(2):16. doi: 10.1038/s12276-019-0214-6 (PMC6376010; doi:10.1038/s12276-019-0214-6)
Supplement: Supplementary file 1 — Supplementary Table 1 [file 12276_2019_214_MOESM1_ESM.docx]

| **Medication** | **HF (LVAD/Explant)**  (n=40) | **HTX**  (n=29) | **p-Value** |
| --- | --- | --- | --- |
| **Tacrolimus [%]** | 0 | 97 | <0.0001 |
| **Everolimus [%]** | 0 | 24 | 0.001 |
| **MMF [%]** | 0 | 83 | <0.0001 |
| **Prednisolone [%]** | 0 | 100 | <0.0001 |
| **Beta-Blocker [%]** | 65 | 28 | 0.007 |
| **ACE-I [%]** | 30 | 24 | 0.79 |
| **Loop Diuretics [%]** | 80 | 62 | 0.11 |
| **MR Antagonists [%]** | 40 | 10 | 0.01 |
| **Statine [%]** | 75 | 93 | 0.06 |
| Abbreviations: ACE-I, angiotensin-converting enzyme inhibitor; MMF, mycophenolate mofetil; MR, mineralocorticoid receptor.  Means ± SD; p-values calculated with unpaired t-test/Mann-Whitney test | | | |

**Supplementary Table 1**
