# Supplementary material for: High-resolution respirometry in human endomyocardial biopsies shows reduced ventricular oxidative capacity related to heart failure
Source: Exp Mol Med. 2019 Feb 14;51(2):16. doi: 10.1038/s12276-019-0214-6 (PMC6376010; doi:10.1038/s12276-019-0214-6)
Supplement: Supplementary file 2 — Supplementary Figure 1 [file 12276_2019_214_MOESM2_ESM.ppt]

## Slide 1
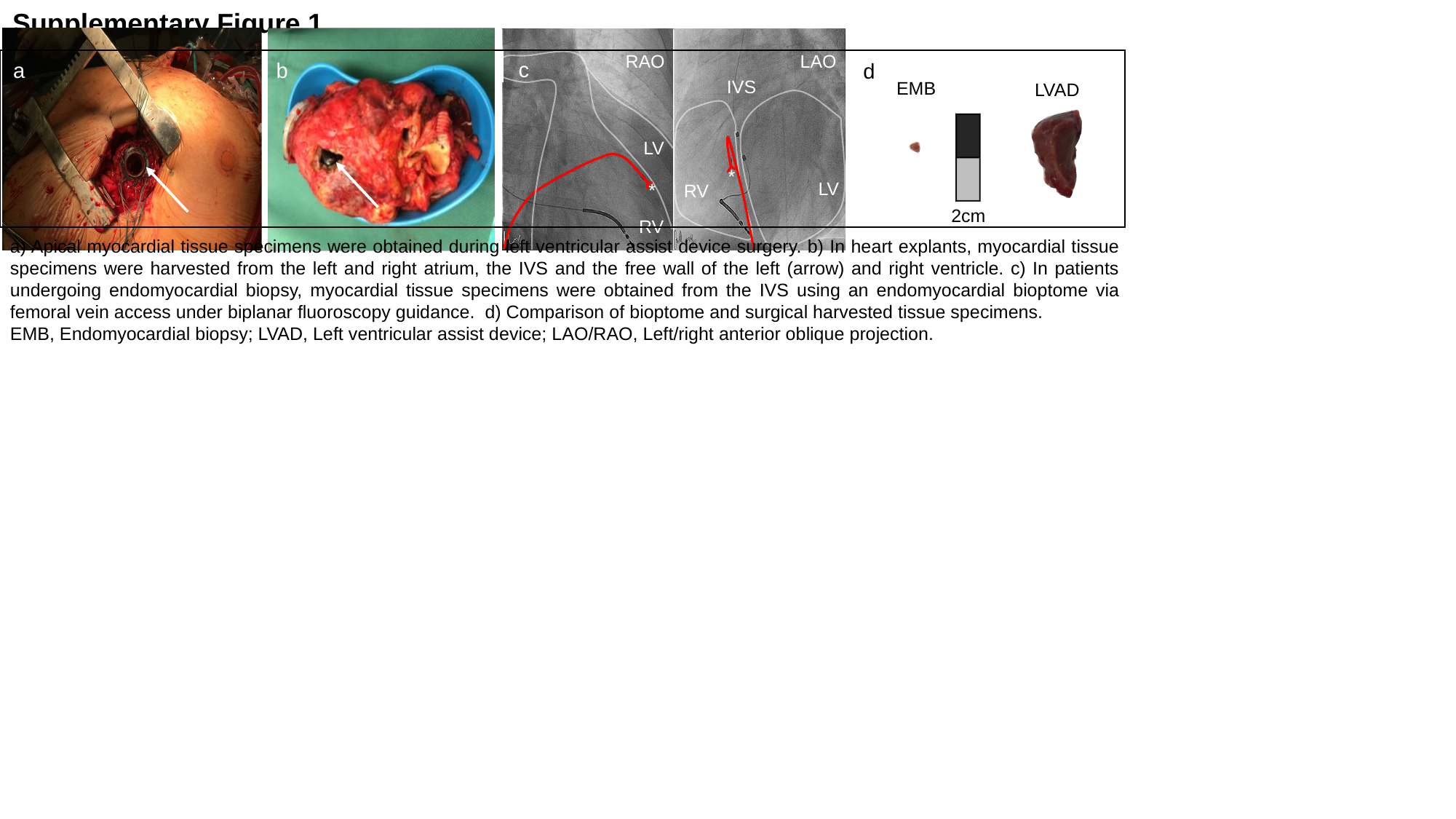

Supplementary Figure 1
IVS
LV
*
LV
*
RV
RV
EMB
LVAD
RAO
LAO
c
a
b
d
EMB
LVAD
2cm
a) Apical myocardial tissue specimens were obtained during left ventricular assist device surgery. b) In heart explants, myocardial tissue specimens were harvested from the left and right atrium, the IVS and the free wall of the left (arrow) and right ventricle. c) In patients undergoing endomyocardial biopsy, myocardial tissue specimens were obtained from the IVS using an endomyocardial bioptome via femoral vein access under biplanar fluoroscopy guidance. d) Comparison of bioptome and surgical harvested tissue specimens.
EMB, Endomyocardial biopsy; LVAD, Left ventricular assist device; LAO/RAO, Left/right anterior oblique projection.
