# Supplementary material for: High-resolution respirometry in human endomyocardial biopsies shows reduced ventricular oxidative capacity related to heart failure
Source: Exp Mol Med. 2019 Feb 14;51(2):16. doi: 10.1038/s12276-019-0214-6 (PMC6376010; doi:10.1038/s12276-019-0214-6)
Supplement: Supplementary file 3 — Supplementary Figure 2 [file 12276_2019_214_MOESM3_ESM.ppt]

## Slide 1
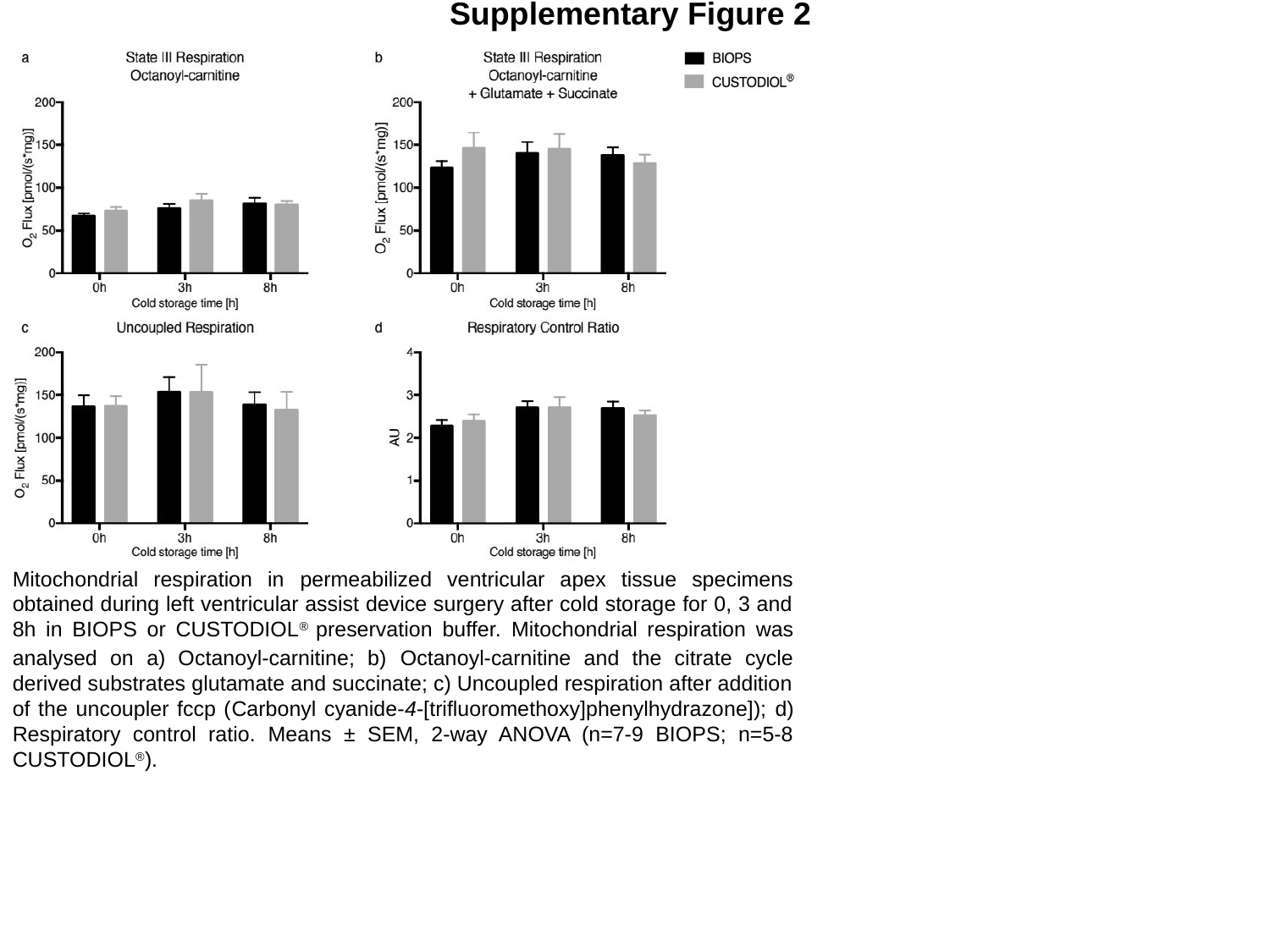

Supplementary Figure 2
Mitochondrial respiration in permeabilized ventricular apex tissue specimens obtained during left ventricular assist device surgery after cold storage for 0, 3 and 8h in BIOPS or CUSTODIOL® preservation buffer. Mitochondrial respiration was analysed on a) Octanoyl-carnitine; b) Octanoyl-carnitine and the citrate cycle derived substrates glutamate and succinate; c) Uncoupled respiration after addition of the uncoupler fccp (Carbonyl cyanide-4-[trifluoromethoxy]phenylhydrazone]); d) Respiratory control ratio. Means ± SEM, 2-way ANOVA (n=7-9 BIOPS; n=5-8 CUSTODIOL®).
