# Supplementary material for: High-resolution respirometry in human endomyocardial biopsies shows reduced ventricular oxidative capacity related to heart failure
Source: Exp Mol Med. 2019 Feb 14;51(2):16. doi: 10.1038/s12276-019-0214-6 (PMC6376010; doi:10.1038/s12276-019-0214-6)
Supplement: Supplementary file 4 — Supplementary Figure 3 [file 12276_2019_214_MOESM4_ESM.ppt]

## Slide 1
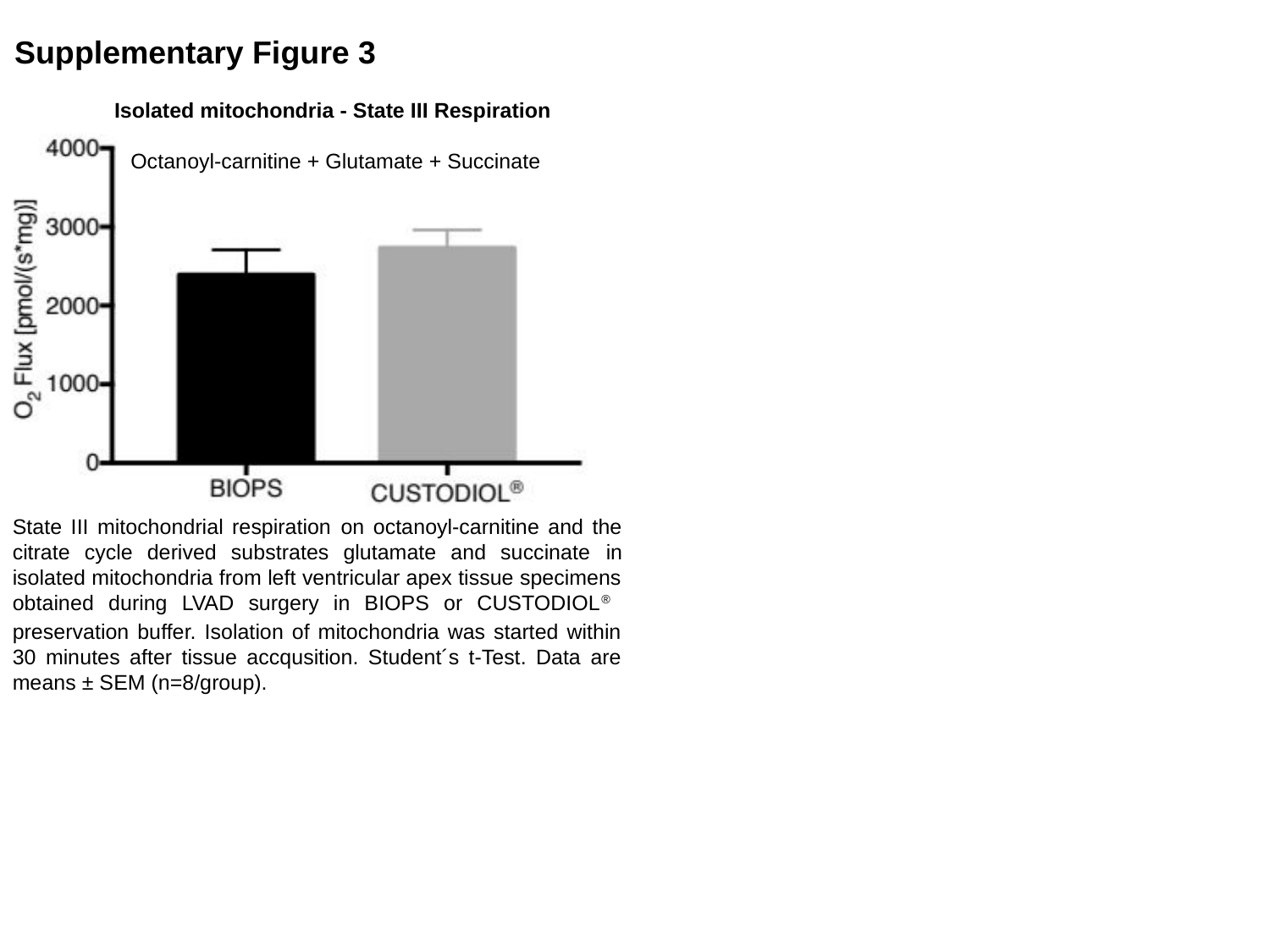

Supplementary Figure 3
Isolated mitochondria - State III Respiration
Octanoyl-carnitine + Glutamate + Succinate
State III mitochondrial respiration on octanoyl-carnitine and the citrate cycle derived substrates glutamate and succinate in isolated mitochondria from left ventricular apex tissue specimens obtained during LVAD surgery in BIOPS or CUSTODIOL® preservation buffer. Isolation of mitochondria was started within 30 minutes after tissue accqusition. Student´s t-Test. Data are means ± SEM (n=8/group).
