# Supplementary material for: High-resolution respirometry in human endomyocardial biopsies shows reduced ventricular oxidative capacity related to heart failure
Source: Exp Mol Med. 2019 Feb 14;51(2):16. doi: 10.1038/s12276-019-0214-6 (PMC6376010; doi:10.1038/s12276-019-0214-6)
Supplement: Supplementary file 5 — Supplementary Figure 4 [file 12276_2019_214_MOESM5_ESM.ppt]

## Slide 1
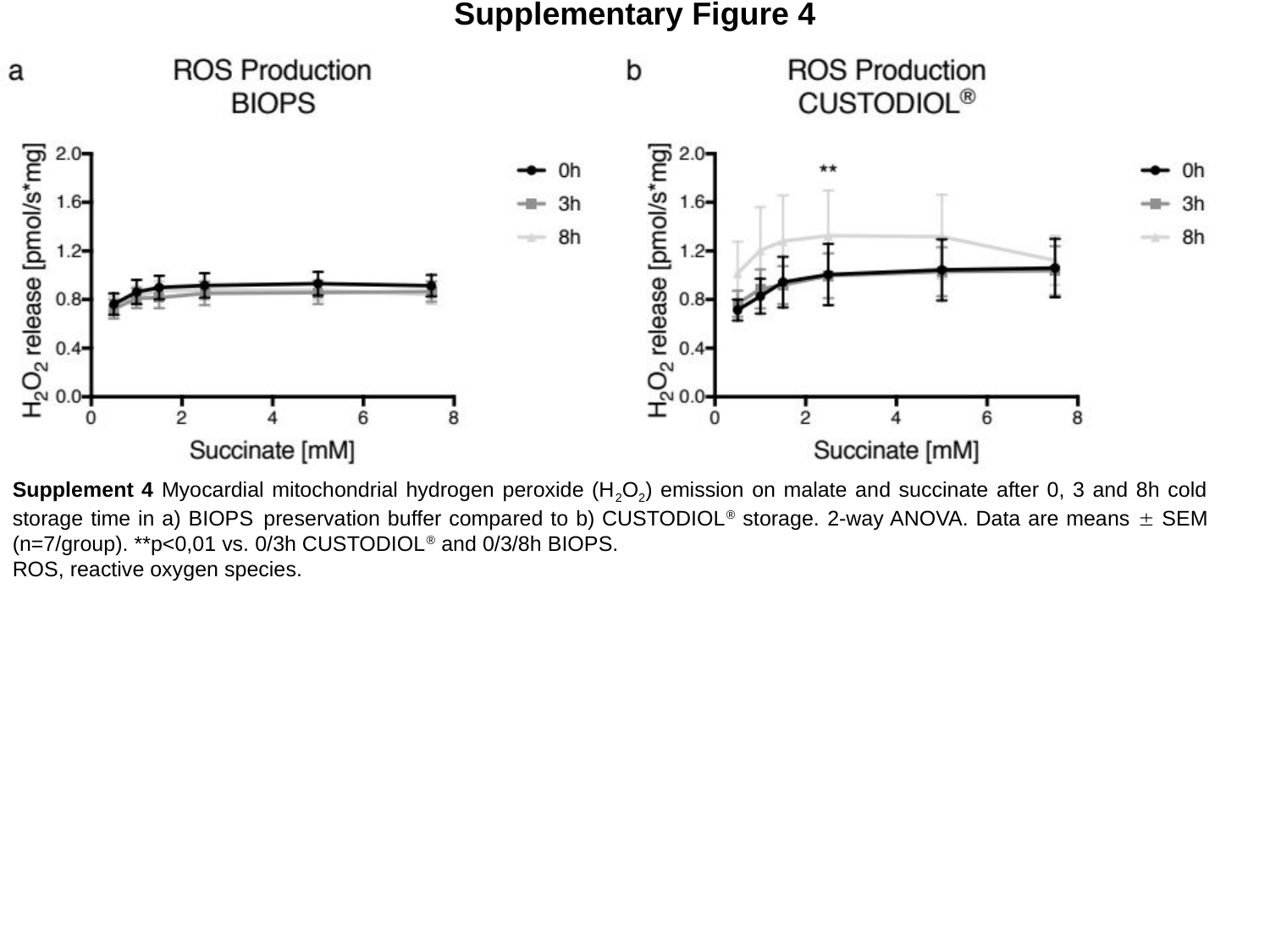

Supplementary Figure 4
Supplement 4 Myocardial mitochondrial hydrogen peroxide (H2O2) emission on malate and succinate after 0, 3 and 8h cold storage time in a) BIOPS preservation buffer compared to b) CUSTODIOL® storage. 2-way ANOVA. Data are means ± SEM (n=7/group). **p<0,01 vs. 0/3h CUSTODIOL® and 0/3/8h BIOPS.
ROS, reactive oxygen species.
